# Restoration of Interaction Between Fatty Acid Oxidation and Electron Transport Chain Proteins In Vitro by Addition of Recombinant VLCAD

**DOI:** 10.3390/biomedicines14010222

**Published:** 2026-01-20

**Authors:** Yudong Wang, Gregory Varga, Meicheng Wang, Johan Palmfeldt, Shakuntala Basu, Erik Koppes, Andrew Jeffrey, Robert James Hannan, Grant Sykuta, Jerry Vockley

**Affiliations:** 1 Department of Pediatrics, School of Medicine, University of Pittsburgh, Pittsburgh, PA 15261, USA; yudong.wang@chp.edu (Y.W.);; 2 Research Unit for Molecular Medicine, Department of Clinical Medicine, Aarhus University, 8200 Aarhus, Denmark

**Keywords:** fatty acid oxidation, mitochondrial electron transfer chain supercomplex (ETC-SC), very-long-chain acyl-CoA dehydrogenase (VLCAD), VLCAD deficiency

## Abstract

**Background/Objectives:** We have previously demonstrated that fatty acid oxidation (FAO) enzymes physically and functionally interact with electron transfer chain supercomplexes (ETC-SC) at two contact points. The FAO trifunctional protein (TFP) and electron transfer flavoprotein dehydrogenase (ETFDH) interact with the NADH^+^-binding domain of ETC complex I (com I) and the core 2 subunit of complex III (com III), respectively. In addition, the FAO enzyme very-long-chain acyl-CoA dehydrogenase (VLCAD) interacts with TFP. These interactions define a functional FAO-ETC macromolecular complex (FAO-ETC MEC) in which FAO-generated NADH^+^ and FADH_2_ can safely transfer electron equivalents to ETC in order to generate ATP. **Methods:** In this study, we use multiple mitochondrial functional studies to demonstrate the effect of added VLCAD protein on mutant mitochondria. **Results:** We demonstrate that heart mitochondria from a VLCAD knockout (KO) mouse exhibit disrupted supercomplexes, with significantly reduced levels of TFPα and TFPβ subunits, electron transfer flavoprotein a-subunit (ETFα), and NDUFV2 subunit of com I in the FAO-ETC MEC. In addition, the activities of individual oxidative phosphorylation (OXPHOS) enzymes are decreased, as is the transfer of reducing equivalents from palmitoyl-CoA to ETC (FAO-ETC flux). However, the total amount of these proteins did not decrease in VLCAD KO animals. These results suggest that loss of VLCAD affects the interactions of FAO and ETC proteins in the FAO-ETC MEC. Reconstitution of VLCAD-deficient heart mitochondria with recombinant VLCAD improved the levels of FAO-ETC MEC proteins and enzyme activities, as well as restoring FAO-ETC flux. It also reduced mitochondrial ROS levels, previously demonstrated to be elevated in VLCAD-deficient mitochondria. In contrast, incubation of VLCAD KO mitochondria with two VLCADs with mutations in the C-terminal domain of the enzyme (A450P and L462P) did not restore FAO-ETC MECs. **Conclusions:** These results suggest that VLCAD is a necessary component of the FAO-ETC MEC and plays a major role in assembly of the macro-supercomplex. These studies provide evidence that both the level of enzyme and its structural confirmation are necessary to stabilize the FAO-ETC MEC.

## 1. Introduction

Very-long-chain acyl-CoA dehydrogenase is a member of a family of enzymes that catalyze the α,β-dehydrogenation of acyl-CoA esters, transferring electrons to oxidative phosphorylation through the electron transfer flavoprotein (ETF) [1,2]. Mutations have been described in numerous patients with VLCAD deficiency, which has now emerged as the most common inborn error of long-chain fatty acid oxidation [3,4,5,6,7,8,9]. In healthy individuals, energy demands during fasting and times of physiologic stress are met through oxidation of stored long-chain fatty acids, which yields reducing equivalents for the mitochondrial respiratory chain and acetyl-CoA for the Krebs cycle [10,11].

Clinical manifestations of LC-FAODs most commonly affect organ systems with high energy demands, such as the heart, skeletal muscle, liver, and brain [5,12,13,14]. Episodes of acute, severe metabolic decompensation often result from periods of elevated energy demand. Baseline rhabdomyolysis and cardiomyopathy often first present in the neonatal period or early childhood and worsen with metabolic decompensation in association with physiologic stressors, including fasting, illness, and exercise. Milder adolescent or adult-onset disease is also recognized. Importantly, chronic, mild energy deficiency can also lead to progressive cardiac dysfunction in mouse models and human patients, even in the absence of catabolic triggers [12,15,16,17,18,19,20,21]. Additional manifestations in TFP/LCHAD deficiencies are believed to stem in part from the accumulation of toxic intermediates of partially metabolized fatty acids, clinically observed as peripheral neuropathy and retinopathy [22,23,24,25,26]. Newborn screening for these disorders reduces but does not eliminate morbidity or mortality [7,13,27,28,29].

Disease management of LC-FAODs typically includes dietary and lifestyle adjustments to minimize the occurrence of metabolic decompensation and associated major clinical events [3,30,31,32,33]. However, the long-term prognosis of patients with LC-FAOD remains poor; one comprehensive study of 187 patients with LC-FAOD reported high rates of associated neurologic symptoms, hypoketotic hypoglycemia, cardiac dysfunction, and mortality, among others [34]. Triheptanoin (containing a seven-carbon backbone), developed in large part by our group, has been approved as the first prescription medication to treat LC-FAOD, providing a new treatment option for patients [32,35,36,37,38,39,40]. While the use of triheptanoin has improved outcomes in LC-FAOD, development of cardiomyopathy and especially elevated baseline rhabdomyolysis with episodic exacerbations continue to be significant problems in treated patients [41,42,43,44]. We and others have previously explored the possible use of gene therapy to treat VLCAD acyl-CoA dehydrogenase (ACAD) deficiencies, but available mouse models of VLCAD deficiency do not adequately reflect human disease and thus further development has stalled [45]. Thus, a large unmet need remains in understanding the pathophysiology of VLCAD and development of new therapies to treat the remaining symptoms.

The coordinated function and physical interaction of three main metabolic pathways is necessary for efficient generation of molecular energy (ATP) in mitochondria: oxidative phosphorylation (OXPHOS), fatty acid β-oxidation (FAO), and the tricarboxylic acid (TCA) cycle [46]. OXPHOS is composed of four enzyme complexes (com I–V) that channel reducing equivalents through complexes I–IV (the electron transport chain; ETC), and generate a voltage gradient across the mitochondrial membrane used by complex V (ATP synthase) to synthesize ATP [46]. Complexes I, III, and IV are organized into a supramolecular structure (“respirasome” or “supercomplexes”) on the inner mitochondrial membrane that increases catalytic efficiency and protects mitochondrial proteins from reactive oxygen species (ROS), intermediates related to the oxidation of molecular oxygen by the ETC [47,48,49,50,51,52]. Electron transfer chain supercomplexes (ETC-SC) are composed of combinations of their component complexes that vary depending on tissue type and metabolic status [53,54,55,56,57,58,59,60].

We have previously demonstrated that LC-FAO enzymes physically and functionally interact with ETC-SCs at two different points [61,62]. The NADH^+^-producing FAO trifunctional protein (TFP) interacts with the NADH-binding domain of com I of the ETC, while electron transfer flavoprotein dehydrogenase (ETFDH) interacts with com III. Additionally, the FAO enzyme very-long-chain acyl-CoA dehydrogenase (VLCAD) physically interacts with TFP, forming a functional FAO-to-ETC macromolecular energy complex (FAO-ETC MEC). This physical contact between FAO and ETC in the FAO-ETC MEC ensures that reducing equivalents (NADH^+^ and Co-QH_2_) generated through FAO reactions are safely transferred through the ETC. Thus, the relationship both protects mitochondrial proteins from exposure to reactive oxygen species and optimizes the kinetics of ATP production. It also optimizes the transfer of the long-chain enoyl-CoA product of the VLCAD reaction to the next FAO reaction, enoyl-CoA reductase of TFP. In this study, we utilize novel in vitro reconstitution studies with purified mitochondria and VLCAD protein to demonstrate that loss of VLCAD in knockout mice has global effects on mitochondrial energy metabolism, demonstrating a critical functional role within the FAO-ETC MEC as well as an enzymatic one.

## 2. Materials and Methods

### 2.1. Institutional Animal Care and Use Committee Assurance

All animal studies were reviewed and approved by the University of Pittsburgh Institutional Animal Care and Use Committee (Protocol IS00026628, “Characterization of ACAD Deficiency Mice”, most recently reapproved 23 April 2025), and animals were maintained in a fully AALAC-accredited animal care facility managed by the University of Pittsburgh Department of Laboratory Animal Research, including on-site veterinary care, following standard housing guidelines. Animals were maintained on a standard chow diet until sacrifice for experiments. All animals were 2–4 months of age and housed with 4–6 animals/cage. Only male animals were studied as they more closely represent the human phenotype, with more severe symptoms than females. The number of animals used in each experiment is noted in the figure legends.

### 2.2. Preparation of Mitochondria from Mouse Heart

Mitochondria were isolated following our previously described protocol and either used immediately for subsequent experiments or stored at −80 °C [61,62]. Briefly, freshly isolated wild-type (WT) and VLCAD knockout (KO) mouse hearts were harvested and immediately homogenized in a tissue blender at high speed for 20 s at 4 °C in a buffer containing 50 mM phosphate, pH 7.5, 100 mM KCL, 0.25 M sucrose, 2.5% glycerol, 1 mM DEAE, and a protease inhibitor mixture (Sigma-Aldrich Co., St. Louis, MO, USA). The homogenates were then centrifuged at 600× *g* for 10 min at 4 °C, and the pellet was discarded. The mitochondria-containing supernatant was subjected to centrifugation at 14,000× *g* for 15 min at 4 °C. The resulting supernatant was discarded, and the pellets were washed once with the homogenization buffer at 14,000× *g* for 15 min at 4 °C. After discarding the supernatant, the pellet was resuspended in the same buffer and stored at −80 °C. Protein concentrations were measured using the DC Protein Assay (Bio-Rad Laboratories, Inc., Hercules, CA, USA). Samples were used at 3 mg/mL of mitochondrial content.

### 2.3. Purification of Expressed VLCAD and SCAD Proteins

Recombinant VLCAD and short-chain acyl-CoA dehydrogenase (SCAD) proteins over-expressed in *E. coli* were purified as previously described [63]. Protein concentrations were measured using the DC Protein Assay (Bio-Rad Laboratories, Inc., Hercules, CA, USA). Enzyme activity was measured with the anaerobic ETF fluorescence reduction assay using a LS50B fluorescence spectrophotometer (PerkinElmer Life Sciences, Shelton, CT, USA). The reaction was started with the addition of the CoA ester substrate to give a final concentration of 25 μm [64].

### 2.4. Reconstitution of VLCAD Knockout Mouse Heart Mitochondria with Purified VLCAD

Wild-type and VLCAD KO mitochondria suspensions were first centrifuged at 14,000× *g* for 15 min at 4 °C. After discarding the supernatant (containing 250 mM sucrose), the mitochondrial pellets were solubilized in 20 mM HEPES, pH 7.4, 100 mM KCL, and 5% glycerol. Samples were diluted with the same buffer to achieve a final protein concentration of 3 mg/mL for each sample. For reconstitution, purified VLCAD was mixed with VLCAD KO mouse heart mitochondria in an amount to equal 1–1.5% of the mitochondrial protein, then incubated at room temperature for 40 min. Samples from wild-type and VLCAD knockout mice were incubated under the same conditions with HEPES buffer.

### 2.5. Blue Native Polyacrylamide Gel Electrophoresis (BN-PAGE)

Mitochondrial samples were prepared and subjected to non-denaturing electrophoresis on native PAGE gels as described [61,62]. Mitochondria were treated with digitonin in a ratio of 1:5–8 (g/g protein/digitonin) and incubated on ice for 20–30 min. The protein concentration was adjusted to 3 mg/mL. The mixture was centrifuged at high speed, and the supernatant was kept. The same amount of protein was loaded across each lane. The BN-PAGE gel was stained with Coomassie blue dye for in-gel protein detection as described [61,62].

### 2.6. In Situ Gel Staining for ETC Enzyme Activity

Gel slices corresponding to the sample wells were excised from the BN-PAGE gel and incubated with reaction buffer systems specific for ETC complex I and V as described [65,66].

### 2.7. BNGE, 2D Electrophoresis, and Western Blotting

A slice of the BN-PAGE gel corresponding to a single sample well was rotated 90 degrees and placed on a 12% SDS-polyacrylamide gel and subjected to electrophoresis for second-dimension separation as described [67]. To visualize the gels, SDS-Western blotting was performed as previously described [61,62].

### 2.8. FAO-ETC Flux Activity Assay

Flux of the substrate between FAO and ETC was performed as previously described [61,62]. FAO acyl-CoA dehydrogenase substrates C20-CoA, palmitoyl-CoA, and octanoyl-CoA were individually used as substrates.

### 2.9. Electron Transfer Chain Complex Activity Assays

ETC complex assays were performed as described [47,61,62].

### 2.10. ETC-SC Reactive Oxygen Species (ROS) Generation Assay

ROS generation by linked FAO-ETC was measured by the fluorescent intensity of 2′,7′-dichlorodihydrofluorescein diacetate (DCFH-DA) as described [68]. Briefly, 30 µg of mitochondrial sample was added to 1 mL of buffer containing 20 mM Tris-HCl, pH 7.4, 100 mM KCl, 5 mM CoQ10, 5 mM NAD, 3 mM ETF, 5 µM DCFH-DA, and 2 mM MgCl_2_ in a glass cuvette. The reaction was initiated by adding 1 mM C16-CoA or C20-CoA. Measurement of the fluorescent intensity was performed with excitation wavelength 485 nm and emission wavelength 520 nm. ROS generation by ETC was measured with the same reaction mixture but using 150 µM NADH to start the reaction.

### 2.11. Reverse-Transcription PCR (RT-PCR) of mRNA Content in WT and VLCAD KO Mouse Heart Extracts

For gene expression analysis by RT-PCR, three control or three VLCAD KO mouse hearts were pooled and mechanically homogenized in RNA Lysis Buffer (Qiagen, Germantown, MD, USA), and mRNA was purified with a mRNeasy purification kit with on-column DNase 1 digestion (Qiagen). First-strand cDNA synthesis was carried out with SSIV Vilo reagent, and RT-PCR was performed with Taq polymerase (Thermo-Fischer/Invitrogen, Waltham, MA, USA). Primers were designed using NCBI Primer Blast (Appendix A Table A1).

### 2.12. Circular Dichroism Spectroscopy

To evaluate the effect of amino acid substitutions on VLCAD structure, two known pathogenic variants identified in patients located to the membrane-binding motif of the enzyme, A450P and L462P, were expressed in *E. coli* and purified as previously described [64]. Purified proteins were analyzed by circular dichroism (CD) spectroscopy using a Jasco^®^ (Easton, MD, USA) J-810 spectropolarimeter equipped with a Peltier temperature controller as previously described [69]. Samples were diluted to a concentration of ~0.5 mg/mL CD in 50 mM Tris buffer, pH 7.4, and CD spectra were measured between 190 nm and 230 nm at 4 °C using a 1 mm pathlength CD-matched quartz cuvette. Data were collected every 0.2 nm with a 1 nm bandwidth. Spectral baselines were corrected by subtracting the CD spectrum of a phosphate buffer blank collected under the same conditions.

### 2.13. Gel Density Measurement and Calculation

In-gel protein density and com I and com V activity stains were quantified using FIJI Software v1.54p for Windows [70].

### 2.14. Statistical Analysis

Data are presented as mean ± standard deviation (SD) for replicates and analyzed with a paired Student’s *t*-test using Prism Graphpad Version 10 (Siemans Digital Industries Software, Boston, MA, USA). This test was selected based on prior experience with data distribution, as determined by visual inspection of multiple distribution plots; however, variance homogeneity was not formally evaluated. Sample numbers are given in the appropriate figure legends.

## 3. Results

### 3.1. Loss of VLCAD Disrupts ETC-SCs in Mitochondria

**Figure 1:**
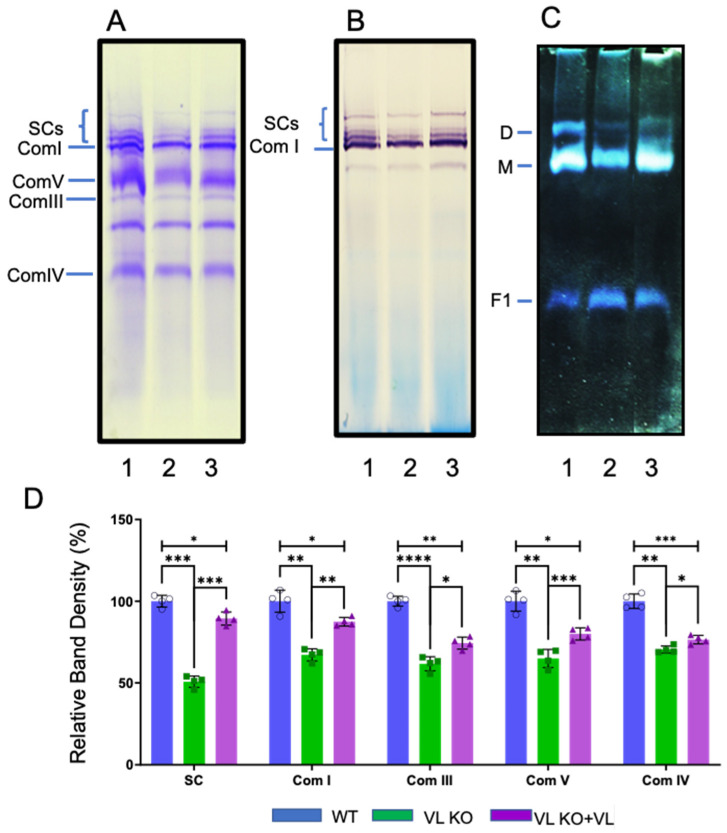
BN-PAGE analysis reconstitution of VLCAD KO mouse heart mitochondria with purified VLCAD. (**A**) BN-PAGE was performed on digitonin-solubilized heart mitochondria from wild-type (WT) and VLCAD KO mouse samples incubated with purified VLCAD (KO + VL), and the gel was stained with Coomassie blue dye. BN-PAGE gels also were stained for com I (**B**) and com V (**C**) activities. (**D**) Band density of the ETC supercomplex (SC) fraction on Coomassie blue-stained gels was quantified using Fiji software and calculated as a percentage relative to WT. In (**C**), dimer, monomer, and F1 are subcomplexes of com V. Uncropped gels are presented in Appendix A Figure A1. * *p* < 0.05; ** *p* < 0.01; *** *p* < 0.001; **** *p* < 0.0001.

Loss of VLCAD in heart mitochondria lead to a reduction in FAO-ETC MECs as demonstrated on BN-PAGE with Coomassie blue staining (Figure 1). The high-molecular-mass FAO-ETC MEC portion of the gel showed a reduction of ~50% compared to samples from wild-type animals (Figure 1A,D), while the levels of individual ETC complexes I–IV were relatively less affected (Figure 1B,D). Incubation of digitonin-solubilized mitochondria from VLCAD KO mice with purified recombinant VLCAD restored the FAO-ETC MEC band to about 90% of WT levels, with recovery of complex I level to near that of WT mitochondria (Figure 1D). These results suggest that the absence of VLCAD protein disrupts the supercomplexes of the FAO-ETC MECs, as well as the individual ETC complexes, though to a lesser extent. Of note, complex V is even more affected than those contained in the FAO-ETC MEC in VLCAD KO mitochondria (Figure 1C), with reduction in dimer and monomer ATPase forms to 25% and 61% of WT, respectively, and an increase in the free F1 fragment by 220%. In mitochondria incubated with purified VLCAD, dimer and monomer forms increased somewhat, while freeform F1 was minimally impacted (Figure 1C,D). Thus, VLCAD also plays a role in the construction, formation, and stabilization of ATPase complexes, especially in its monomer form.

### 3.2. VLCAD and Other FAO Proteins Are Reassembled into Supercomplexes in VLCAD KO Mouse Heart Mitochondria When Incubated with Recombinant VLCAD

**Figure 2:**
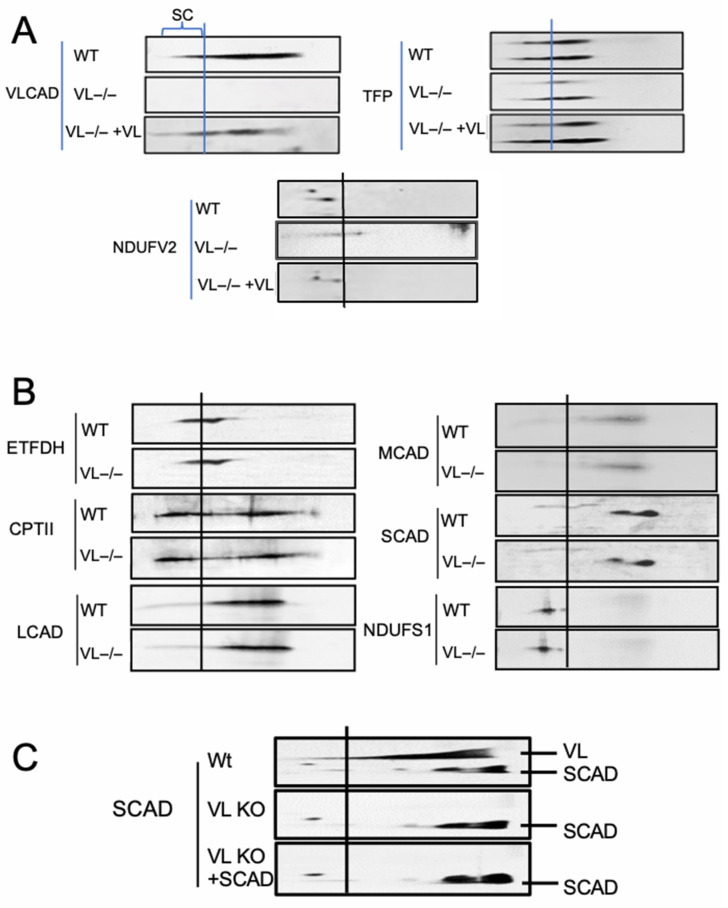
VLCAD and other long-chain FAO proteins are reassembled into ETC-SC MECs via incubation with purified recombinant VLCAD. (**A**) VLCAD, TFPα, TFPβ, ETFα, and com I NDUFV2 were significantly reduced in VLCAD KO mouse heart mitochondria as demonstrated by Western blots of 2D BN-PAGE gels. Incubation of VLCAD KO samples with purified recombinant VLCAD resulted in recovery of all four proteins in ETC-SCs. (**B**) FAO proteins ETFDH, CPTII, LCAD, MCAD, SCAD, and ETC complex I NDUFS1 subunit antibody were unchanged in VLCAD KO mitochondria compared to WT. (**C**) Incubation of VLCAD KO heart mitochondria with purified recombinant SCAD protein did not affect FAO protein with ETC-SCs. The vertical line in each blot represents the location of isolated ETC com I. Thus, proteins located to the left of this line are associated with ETC-SCs. Uncropped gels are presented in Appendix A Figure A2, Figure A3 and Figure A4.

To further characterize FAO protein interaction with ETC-SCs, WT and VLCAD KO mouse mitochondria were separated by 2D BN-PAGE after incubation with purified recombinant VLCAD. Western blots of these gels showed that, as expected, VLCAD was not present in VLCAD KO samples (Figure 2A). In addition, the TFPα, TFPβ, ETFα, and complex I NDUFV2 signals were reduced compared to WT mitochondria, especially in the region of the gel corresponding to ETC-SCs. When incubated with purified recombinant VLCAD, these proteins significantly increased both in the SC portion of the gel and overall when including the unbound portion of each protein (Figure 2A). However, not all FAO and ETC proteins were affected in VLCAD KO mitochondria. FAO proteins ETFDH, CPTII, LCAD, MCAD, SCAD, and ETC complex I subunit NDUFS1 are present in nearly the same amount as in WT mitochondria (Figure 2B). As a negative control, incubating VLCAD KO mitochondria with purified recombinant SCAD (which has high homology to VLCAD) had no effect on ETC-SCs or their interaction with long-chain FAO proteins (Figure 2C).

### 3.3. Flux of Substrate from FAO to ETC and Generation of ROS

**Figure 3:**
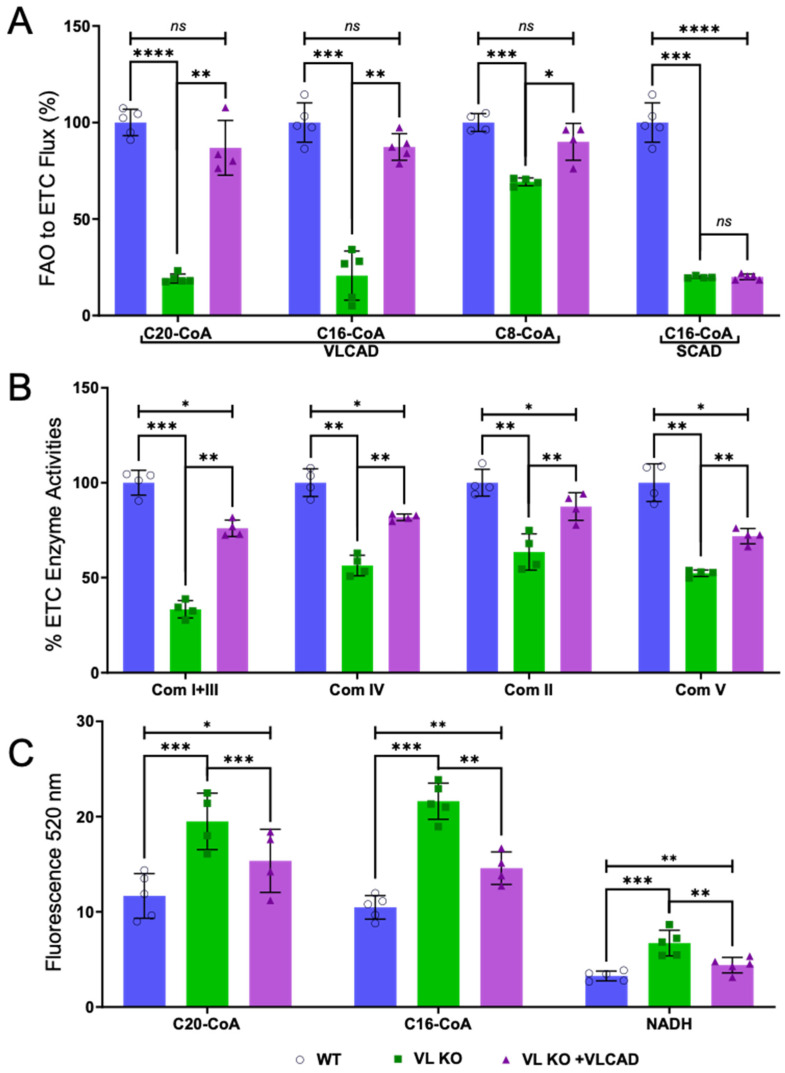
FAO-ETC flux, ETC complex activities, and ROS generation in WT and VLCAD KO mitochondria. (**A**) Flux of reducing equivalents from FAO to ETC in digitonin-permeabilized mitochondria from wild-type (WT) and VLCAD knockout (VL) mitochondria; VL KO + VL mice heart mitochondria were measured with different fatty acid substrates. The FAO-ETC flux activity of WT and VL KO compared to VL KO recombined with SCAD is also shown. *N* = 3–5. (**B**) ETC enzyme activities of WT, VL KO, and VL KO + VL mice heart mitochondria were measured with different substrates (complex I + III, complex V, complex IV, complex II). (**C**) ROS generation assays for WT VL KO and VL KO + VL were conducted in the presence of FAO substrates (C20-CoA, C16-CoA) and NADH^+^, a complex I substrate, respectively. *N* = 3–5. All changes between WT and VLCAD KO mitochondria were statistically significant. * *p* < 0.05; ** *p* < 0.01; *** *p* < 0.001; **** *p* < 0.0001, ns—not significant. Uncropped gels are presented in Appendix A.

The integrity of the FAO-ETC MEC was examined further in VLCAD KO mitochondria through assays measuring flux of reducing equivalents from FAO to ETC and across ETC. (Figure 3A). Here, incubation of digitonin-permeabilized mitochondria with long-chain FAO substrates (C20- or C16-CoA) showed a significantly decreased (to ~20% of WT) reduction in cytochrome c in mitochondria from KO compared to WT mice. In contrast, flux from the medium-chain substrate C8-CoA to cytochrome c was only slightly impaired (to ~70% of WT). Incubation of VLCAD KO mitochondria with purified recombinant VLCAD led to recovery of up to 90% of flux from FAO to cytochrome c compared to WT in the presence of long-chain FAO substrates C20CoA and C16CoA. As a negative control, incubation with purified SCAD showed no effect on flux activity with C16-CoA as a substrate. In addition, ETC complex enzyme activities were reduced in KO compared to WT mitochondria to between ~30% to ~60% of WT levels, and were partially restored (to about ~52% to ~78% of WT levels by incubation with purified recombinant VLCAD (Figure 3B)). These results indicate that VLCAD is essential for forming or stabilizing the FAO-ETC MEC structure and function.

Mitochondria generate reactive oxygen species (ROS) in the form of superoxides as a function of normal oxidative phosphorylation. However, ROS is increased in primary defects in ETCs, probably due to leakage from compromised ETC-SCs [62,71,72]. We have shown increased ROS production in fibroblasts from VLCAD-deficient patients [73]. To examine this phenomenon further, we measured ROS levels in intact VLCAD KO compared to WT digitonin-permeabilized mitochondria (Figure 3C). ROS production increased significantly to 1.6~2.1 times that of WT when incubated with the long-chain FAO substrates C20- and C16-CoA, respectively. This increase was blunted when the mitochondria were incubated first with purified recombinant VLCAD. It was reduced to 1.2 and 1.1 times that of WT. A similar pattern of changes was seen when the mitochondria were incubated with the ETC complex I substrate NADH, though the level of ROS produced was lower than with the FAO substrates. Note that the level of ROS generated in the presence of FAO substrates was much higher (2.7 and 3.2 times) compared to NADH in WT mitochondria. These results indicate that in normal fibroblasts, FAO generates more ROS than ETC, a tendency that was exaggerated in VLCAD KO mitochondria.

### 3.4. Transcription of FAO and ETC Proteins Is Unaffected in VLCAD KO Mice

**Figure 4:**
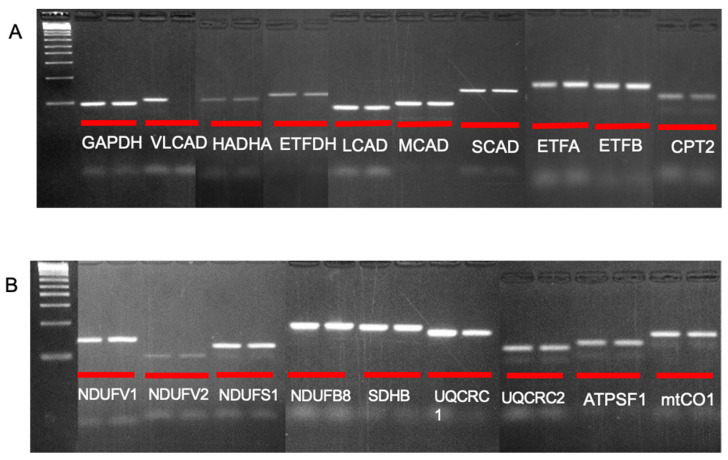
Analysis of FAO and ETC subunit mRNA levels in WT and VLCAD KO mouse heart. mRNA was extracted from WT and VLCAD KO mouse hearts and reverse-transcribed, and cDNA of various FAO and ETC proteins was examined by PCR followed by agarose gel electrophoresis. The labels indicate gene designations for each gel slot. GAPDH was used as an amplification and loading control. (**A**,**B**) show separate gels for the indicated gene primer sets. Uncropped gels and gene abbreviations are presented in Appendix A Figure A5 and Figure A6.

To assess the impact of VLCAD KO on transcription of genes for the FAO and ETC proteins above, mRNA was extracted from WT and VLCAD KO mouse hearts and analyzed by RT-PCR (See Appendix A Table A1 for a list of all genes. As expected, VLCAD KO mice had no identifiable VLCAD message (Figure 3A). Amplification of message for HADHA, ETFDH, CPTII, ETFα, ETFβ, CPTII, LCAD, MCAD, and SCAD (Figure 4A); ETC complex I subunits NDUFS1, NDUFV1, NDUFV2, and NDUFB8; ETC complex III subunit UQCRC2; complex II subunit SDHB; complex IV subunit mtCQ1; and complex V subunit ATP5S1 (Figure 4B) was qualitatively unchanged in VLCAD KO compared to WT animals. Only mRNA for complex III subunit UQCRC1 was decreased. These results confirm that the reduction in protein content in the VLCAD KO mitochondria FAO-ETC MECs (Figure 1) is not due to transcriptional changes, but likely complex instability with subsequent degradation of the component proteins.

### 3.5. Reconstitution of FAO-ETC MEC Complexes with Variant VLCAD Proteins

**Figure 5:**
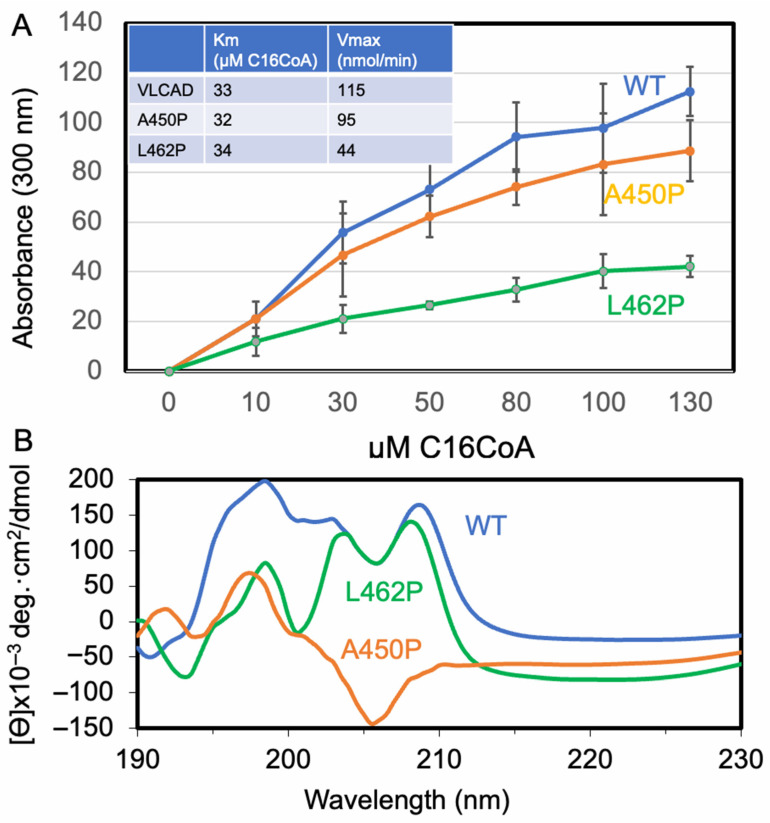
Enzyme activity and CD spectra of purified normal and A450P and L462P mutant VLCADs. The enzymes were expressed in *E. coli* and purified to homogeneity. (**A**) CD spectra from wavelength 190–230 nm. (**B**) Enzyme activity as measured with the ferricenium reduction assay using palmitoyl-CoA as substrate. Assay time curves are shown in the main graph and calculated catalytic parameters are presented in the table insert.

VLCAD contains a membrane-binding domain at its carboxyl terminus that is distinct from MCAD and SCAD, which are both soluble enzymes in the mitochondrial matrix [63]. We have previously demonstrated that mutations in this domain affect binding to mitochondrial membranes [64]. However, their effect on interaction with the FAO-ETC MECs has not been examined. To do so, normal human VLCAD and two such mutations identified in patients with VLCAD deficiency, A450P and L462P, were expressed in *E. coli* and purified as previously described [64]. As expected, the Vmax of the recombinant mutant enzymes, as measured with the ferricenium reduction activity with palmitoyl-CoA as substrate, was decreased compared to normal VLCAD, while their Km values were similar (Figure 5A). The CD spectra of purified mutant enzymes were altered compared to normal enzyme, consistent with significant changes in their secondary structure (Figure 5B). Specifically, the A450P and L462P proteins demonstrated decreased ellipticity in the 190-to-200 nm region of the spectrum compared to the normal enzyme. L462P also showed a significant decrease in ellipticity at 202–210 nm, while A450P had minimal change in that region. A450P and L462P both had reduced ellipticity at 220 nm compared to the control enzyme, indicating disruption of α-helices in the mutant proteins. In total, these data suggest that both mutant enzymes show an altered secondary structure compared to the control [74,75]. The kinetic parameters of the purified control and mutant VLCADs showed a much greater reduction in Vmax for the L462P enzyme than for the A450P variant, consistent with previous findings on the two mutant enzymes [64].

### 3.6. Incubation of Mitochondria from VLCAD-Deficient Mitochondria with Purified A450P and L462P VLCAD Variant Proteins

**Figure 6:**
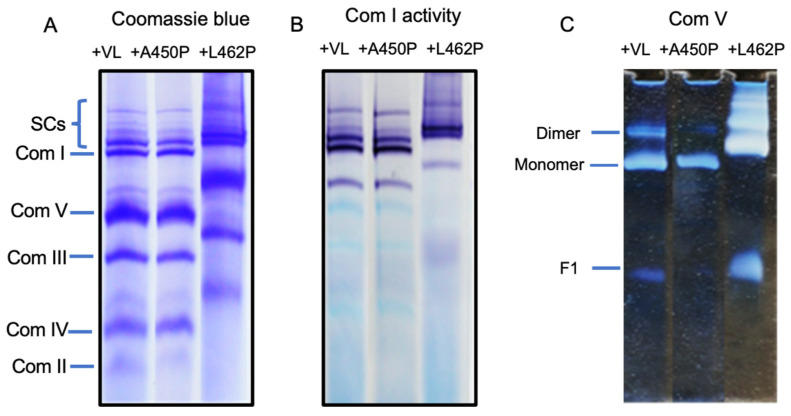
BN-PAGE analysis of VLCAD-deficient heart mitochondria with purified recombinant control and mutant A450P and L462P VLCAD. (**A**) The gel was stained with Coomassie blue dye (**A**). The expected migration of the respiratory chain individual and supercomplexes is marked on the left. Duplicate gels were stained with an in situ com 1 activity stain (**B**) and com V activity stain (**C**). Migration of complex V dimers, monomers, and F1 subunits are marked to the left of the gel.

To examine the effect of the C-terminal-variant VLCAD proteins on the FAO-ETC MECs, digitonin-solubilized mitochondria from VLCAD KO mice were incubated with purified control or variant VLCAD and then examined via BN-PAGE. As seen in Figure 1, VLCAD KO mitochondria have decreased ETC-SC content and loss of com V dimers, which is rescued by the addition of purified control VLCAD. Incubation instead with purified A454P VLCAD similarly led to rescue of the SCs (Figure 6A,B), but com V dimers were still absent (Figure 6C).

In contrast, incubation of VLCAD KO mitochondria with purified L462P VLCAD protein demonstrated an increase in molecular mass of ETC-SCs and com I, along with persistent loss of monomeric and dimeric com V, with an increase in F1 (Figure 6A–C). In addition, four high-molecular-weight bands with com V activity, along with an upward shift in the other complex bands identified with Coomassie blue staining, were present. These results suggest that the A450P protein maintained near-normal interactions with ETC proteins, while the mutant VLCAD protein was non-specifically binding to the various ETC complexes and supercomplexes.

**Figure 7:**
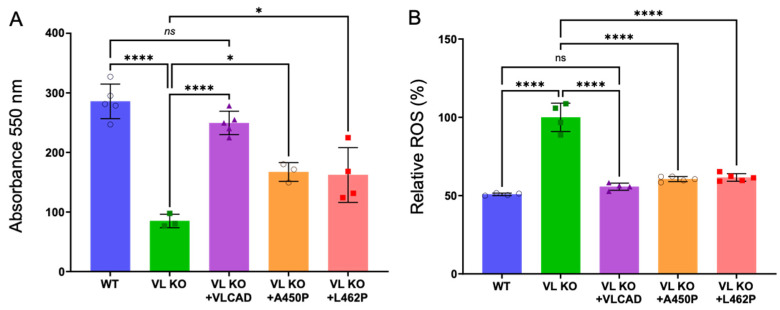
The effect of incubation of VLCAD-deficient mitochondria with purified recombinant control and A450P and L462P mutant VLCAD on coupled FAO-ETC flux activity, ETC complex activities, and ROS generation as measured with DCR-DA. (**A**) shows the percent recovery of flux of reducing equivalents from FAO to ETC with palmitoyl-Co used as substrate, as measured by the increase in absorbance at 550 nm. (**B**) shows the percentage of ROS levels in mitochondria in VLCAD KO mitochondria incubated with mutant vs. control VLCAD. (*N* = 4–6 for all assays as described in the methods). * *p* < 0.05; **** *p* < 0.0001, ns—not significant.

As seen in Figure 3, the flux of reducing equivalents from palmitoyl-CoA to the ETC was reduced in permeabilized VLCAD KO mitochondria, as were ETC complex activities. Addition of purified recombinant VLCAD to permeabilized VLCAD KO mitochondria improved linked flux to nearly 90% of the activity in control mitochondria. However, the addition of purified recombinant A450P and L462P to VLCAD KO mitochondria increased FAO-ETC-coupled flux by only 58.1% and 52.5%, respectively, compared to incubation with control VLCAD (Figure 7A); these values were proportional to their enzyme activity.

ROS generation in VLCAD KO mitochondria was significantly higher than in mitochondria from wild-type mice, but it was nearly normalized when the VLCAD KO mitochondria were incubated with purified control and mutant recombinant VLCAD protein (Figure 3C). This suggests that the FAO-ETC MECs were at least partially stabilized by stable VLCAD protein, even if they had reduced activity. However, ROS generation following reconstitution of VLCAD KO mitochondria with purified A450P and L462P was higher than with the native enzyme (Figure 7B). The increase in ROS generation could be due in part to electron leak from ETC supercomplexes induced by the mutant enzymes.

**Figure 8:**
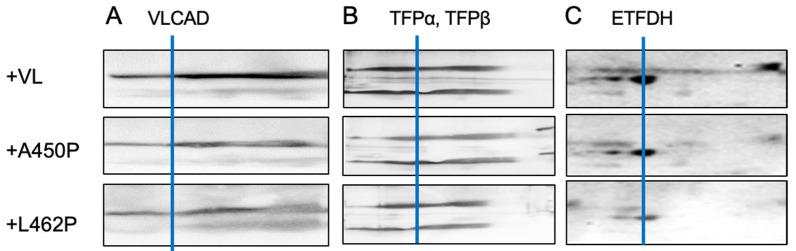
Effect of reconstitution of VLCAD KO mitochondria with wild-type and variant A450P and L462P VLCAD proteins on FAO-ETC MECs. Two-dimensional gels with BN-PAGE as the first dimension followed by SDS-PAGE in the second dimension were subjected to Western blot analysis to examine the effect of the purified VLCAD proteins on FAO and ETC protein migration. Detection antibodies included FAO protein antibodies (**A**) anti-VLCAD, (**B**) anti-TFPα and TFPβ, and (**C**) anti-ETFDH. ETC-SCs are located to the left of the blue line in each panel.

To further assess FAO-ETC MEC stability, VLCAD KO mitochondria incubated with purified recombinant VLCAD were examined by BN-SDS PAGE followed by Western blotting. Incubation with purified control VLCAD protein largely restored migration of FAO proteins (VLCAD, TFP α and β, and ETFDH) to the ETC-SC portion of the non-denaturing gel. Incubation with the A450P and L462P VLCADs also restored migration of FAO proteins to the high-molecular-mass portion of the non-denaturing gel, though slightly less efficiently than the control enzyme in some cases (Figure 8). These results confirm the results of Figure 7, and highlight the importance of VLCAD in the stability of the FOA-ETC MEC.

## 4. Discussion

### 4.1. VLCAD Plays an Important Role in the Assembly and/or Stability of the FAO-ETC MEC

We have previously shown that FAO and ETC proteins interact to form a multi-enzyme complex (MEC) that optimizes catalysis of substrates and transfer of reducing equivalents from FAO to ETC enzymes [61,62]. In this model, the TFPα subunit directly interacts with the matrix arm of ETC com I, and mutations in *HADHA* affecting the stability of TFP are also predicted to impact the stability of the FAO-ETC MEC. The model also shows that VLCAD interacts with TFP, allowing substrate channeling. However, the effect of mutations on the VLCAD protein on the FAO-ETC MEC structure is less straightforward to predict. To examine this latter question, we examined the effect of incubation of permeabilized mitochondria for VLCAD knockout mice with purified, recombinant control and mutant VLCAD proteins. The VLCAD KO mutant mitochondria demonstrated significantly reduced levels of ETC supercomplex forms 1–3 on BN-PAGE, along with reductions in all individual ETC complexes, suggesting the role of VLCAD in FAO-ETC MEC assembly and/or stability. Incubation of the KO mitochondria with control VLCAD led to recovery of the individual complex and supercomplex proteins (including NDFUV2, a key com I protein that interacts with TFP), indicating that VLCAD can reassemble with and stabilize the FAO-ETC MEC in VLCAD-deficient mitochondria. Additionally, these results support our previous identification of the FAO-ETC MEC as a naturally existing and stable structure that connects FAO and the ETC both physically and functionally [12,13].

### 4.2. The FAO-ETC MEC Mediates Functional Linkage of FAO and ETC

We previously demonstrated transfer of reducing equivalents from FAO to ETC using a coupled flux assay in intact mitochondria that measured activity of ETC com III (cytochrome c reduction at 550 nm) following incubation with long-chain FAO substrates [62,76]. The overall reaction occurred with the same catalytic efficiency as purified VLCAD, demonstrating substrate channeling across the FAO-ETC MEC. Indeed, in this study, VLCAD KO mitochondria exhibited dramatically reduced FAO-ETC flux activity (~20% of WT) in the presence of long-chain substrates, as well as moderately decreased individual ETC complex activities. Incubation of VLCAD KO mitochondria with purified, recombinant control VLCAD protein led to significant recovery of these activities and ETC com I protein. Com I + III activity, originally reduced in VLCAD KO mitochondria, was increased upon incubation with VLCAD. The com I protein NDUFV2 is the contact point between FAO and ETC (through TFP and com I). Its reduction in VLCAD KO mitochondria and restoration upon incubation with control VLCAD protein suggests that the interaction of VLCAD and TFP is necessary for stabilizing the binding of TFP with com I.

### 4.3. The Absence of VLCAD Protein Affects ETC Com I and V Activity

As noted above, in-gel Coomassie blue and com I activity staining of blue native polyacrylamide gels were significantly reduced in VLCAD-deficient mitochondria (Figure 1). This reduction was observed in both supercomplex and com I bands. Incubation of the deficient mitochondria with purified recombinant control VLCAD protein restored them to levels near those seen in wild-type mitochondria. These results offer further support for the structural role of VLCAD in the FAO-ETC MEC. Additionally, com V activity staining identified a loss of the mature dimer form of com V in VLCAD-deficient mitochondria that is restored upon incubation with purified recombinant VLCAD protein. This finding was unexpected as com V is not known to interact directly with ETC-SCs, suggesting an indirect effect on com V stability. The dimer of com V is located in the cristae of the inner mitochondrial membrane. The shape and function of mitochondrial cristae have previously been linked with the assembly and stability of ETC-SCs [77,78,79,80]. Since incubation of VLCAD-deficient mitochondria with VLCAD led to partial recovery of com V dimers, we hypothesize that the loss of integrity of the FAO-ETC MEC disrupted the cristae structure, leading to secondary loss of com V stability. Additional imaging studies will be necessary to further investigate this possibility. Of note, our results provide a basis for understanding our previous report of decreased mitochondrial ATP production in VLCAD-deficient fibroblasts [73].

### 4.4. ROS Generation in VLCAD-Deficient Mitochondria

The primary site of ROS generation in the mitochondria is the ETC, and while physiologic levels are essential, increased levels become pathologic [81,82,83]. Indeed, one of the main theoretical benefits of ETC supercomplexes is protection of the mitochondrial matrix components from ETC reaction products, including mitochondrial superoxides [84,85]. Disruption of this structure is a primary reason for increased ROS in disorders of the mitochondrial ETC. Generation of ROS by FAO has also been described, but it is not clear if it arises directly from this pathway or if it results from secondary ETC dysfunction [73,86,87,88,89]. We have hypothesized that mutations in VLCAD disrupted the integrity of the FAO-ETC MEC, leading to leakage of ROS from the complex, thus increasing its concentration in the mitochondrial matrix. Our current studies support this hypothesis. Incubation of long-chain saturated FAO substrates or NADH^+^ (an ETC com I substrate) with VLCAD KO mitochondria led to increased ROS production compared to wild-type mitochondria. Additionally, incubation of VLCAD KO mitochondria with purified recombinant VLCAD rescued this phenomenon, confirming that an appropriately assembled FAO-ETC MEC is necessary for the management of ROS levels in mitochondria. This aligns with the observation that in VLCAD KO mitochondria, substrate flux from FAO to ETC is dramatically reduced (Figure 3) with the concomitant increase in ROS (Figure 7), emphasizing the physiologic relevance of the FAO-ETC MEC structure. Note that DCF-DA does not directly detect superoxide or H_2_O_2_ but rather is an “oxidative stress marker” [90], though in the current studies, the signal can only be mitochondrially generated, as all in vitro manipulations occurred after isolation. Additionally, it should be noted that ROS generation in wild-type mitochondria incubated with FAO long-chain substrates is more than three times higher than with NADH^+^ (Figure 3C). The energetic efficiency of mitochondrial fatty acid oxidation (FAO) is exemplified by the fact that a single molecule of C16-CoA can generate up to 106 ATP, far surpassing the yield from NADH oxidation alone. This underscores the critical importance of maintaining functional coupling between LC-FAO enzymes and the ETC. Disruption of these interactions not only impairs energy production but also leads to excessive reactive oxygen species (ROS) generation. ROS elevation, originating upstream of the ETC, could exert more profound clinical consequences than ETC dysfunction itself. Therefore, restoring FAO-ETC coupling offers a promising therapeutic strategy to mitigate oxidative stress and its downstream pathologies.

### 4.5. Co-Assembly

The assembly of ETC complexes (especially com I) and ETC-SCs has been well described [91,92,93,94,95]. In VLCAD KO mitochondria, we found that the loss of VLCAD protein disrupts the formation of SCs, while reconstitution of VLCAD-deficient mitochondria with purified VLCAD results in the recovery of FAO-ETC MECs. Moreover, knockout of VLCAD affects only a select group of FAO proteins and ETC complexes, as was apparent in both the FAO-ET MEC and lower-molecular-mass individual protein and ETC complex structures (Figure 2). Given that mRNA RT-PCR studies were consistent with normal transcription of the genes for these proteins, and that most of the proteins were present in the overall amounts in VLCAD mitochondria, we conclude that the reduced FAO-ETC MEC content in VLCAD-deficient mitochondria is due to impaired assembly or stability, and that the individual proteins can reassemble when the mitochondria are provided with exogenous VLCAD protein. These results indicate that VLCAD plays an important role in assembling and/or stabilizing supercomplexes. Specifically, we conclude that VLCAD likely functions together with TFP, ETF, and NDUV2 to stabilize FAO-ETC MECs, defining a functional assembly group of proteins. We suggest the term “VLCAD co-assembly proteins” for this phenomenon. From our results, we can conclude that the VLCAD co-assembly proteins include at least both FAO and ETC com I proteins. However, it is likely that this is an incomplete list of “co-assembly proteins” and further investigation is necessary to determine the precise role of VLCAD in FAO-ETC MEC assembly. Elucidating the molecular basis of this co-assembly will provide critical insight into the mechanistic links between FAO and ETC dysfunction in disease states, with direct implications for the development of improved diagnostic strategies and targeted therapeutic interventions.

### 4.6. The Effect of VLCAD Mutations on FAO-ETC MEC Assembly

Genetic disorders involving ETC complex and supercomplex assembly are an essential subgroup of mitochondrial respiratory chain deficiencies with poor therapeutic options available [96]. Additionally, defective assembly and dysfunction of mitochondrial ETC-SCs is a secondary hallmark of multiple medical conditions, while increased supercomplex formation in humans is an adaptive mechanism for increased energy demand [95,97,98]. Given the role we have now described for VLCAD in the FAO-ETC MEC, we anticipated that mutations in VLCAD would also affect this complex. VLCAD mutations A450P and L462P identified in patients with VLCAD deficiency are located in the C-terminal inner mitochondrial membrane-binding domain of VLCAD [64]. Indeed, we found that these variant proteins were less effective in inducing reassembly of the FAO-ETC MEC in VLCAD-deficient mitochondria, leaving persistent elevations in ROS and impaired linkage of FAO-to-ETC function. Their effects in reconstitution studies were not identical, consistent with the structural differences identified in the purified recombinant proteins, as well as in residual enzyme activity and CD structure. Overall, the L462P variant is functionally more impaired than the A450P variant, although both patients exhibit a similarly mild presentation [64].

### 4.7. Relationships Between NADH-Generating Proteins and Complex I of ETC-SC

NADH^+^ is typically used as a com I substrate (NADH^+^ dehydrogenase) and to characterize the integrity of flux through the ETC supercomplexes (com I–III activity as shown by reduction in cytochrome c). Here, we demonstrate that the long-chain FAO substrates are more robust sources of reducing equivalents for com I by way of NADH^+^ generated by the long-chain 3-hydroxyacyl-CoA dehydrogenase activity. Our hypothesis is that, in addition to this FAO activity, many other NADH^+^-generating enzymes in mitochondria could interact with ETC-SCs, including pyruvate dehydrogenase complex (PDH), malate dehydrogenase (MDH2), α-ketoglutarate DH (a-KGDH), short-chain 3-hydroxyacyl-CoA dehydrogenase (SCHAD), and branched-chain alpha-keto acid dehydrogenase complex (BCKDH). Given our results, we speculate that these enzymes might also play a role in forming and stabilizing multi-enzyme complexes with ETC supercomplexes.

## 5. Conclusions

It has long been recognized that many of the enzymes identified in the mitochondrial matrix are organized into functional catalytic pathways. Our findings continue to demonstrate that the architecture of the mitochondrial matrix is not random. Rather, proteins of related pathways (here, FAO and ETC) physically interact. Furthermore, we have now shown that these multi-enzyme complexes exhibit greater global secondary instability induced by the loss or alteration of individual components. Ultimately, it is reasonable to predict that the network of physical interactions will extend far more than has been experimentally shown so far, generating a mitochondrial matrix architecture that is critical for normal physiologic homeostasis. Future studies of inborn errors of mitochondrial metabolism and their treatment should consider such interactions and the disruption to the broader metabolome that they predict.

## Figures and Tables

**Figure A1:**
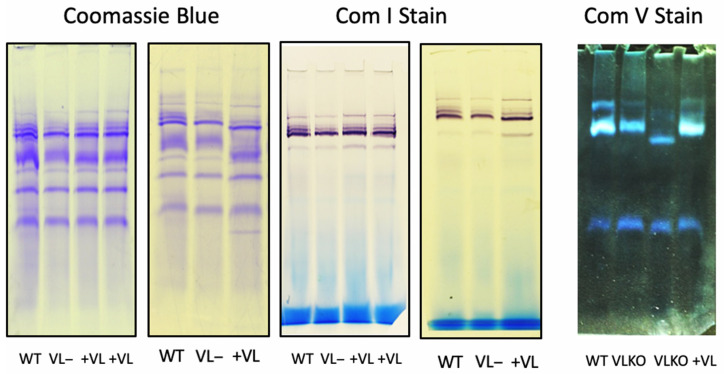
Uncut gel photos used to measure data points in Figure 1. The first two panels were stained with Coomassie blue dye. The second two panels were stained with an ETC complex I stain. The fifth panel was stained with a complex V stain. Lanes 1, 2, and 4 were used for the figure.

**Figure A2:**
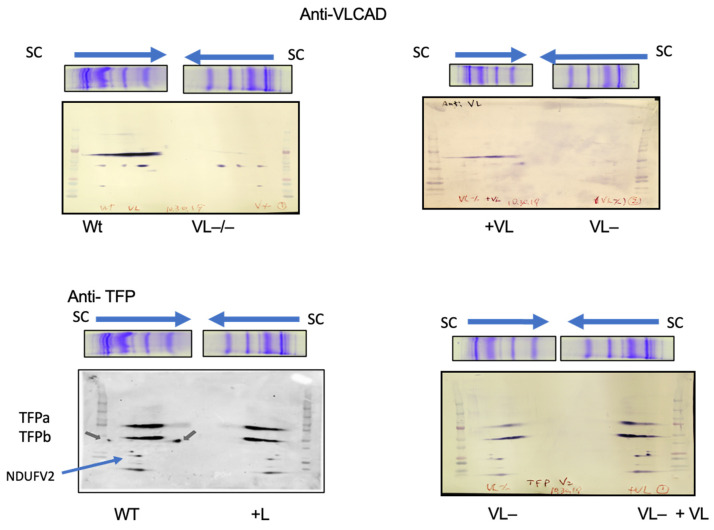
Uncut gel photos used for Figure 2 as marked. Note that the strips from the first-dimension blue native gel were laid in opposite orientation for the SDS-PAGE dimension. The blue arrow indicates the main protein band selected as the center of the Figure 2 panel.

**Figure A3:**
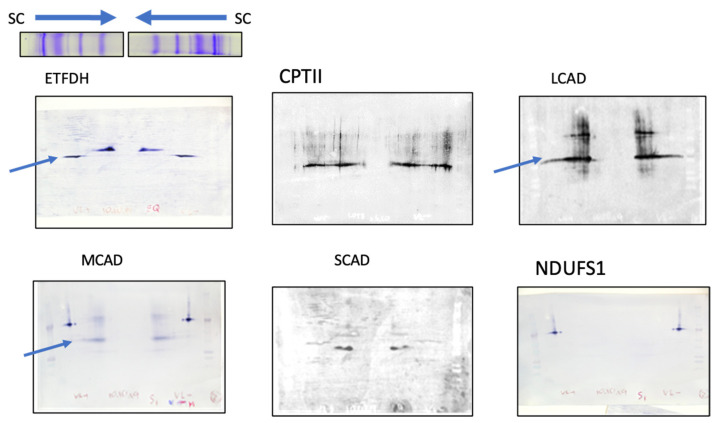
Additional uncut gel photos used for Figure 2 as marked. The blue arrow indicates the main protein band selected as the center of the Figure 2 panel.

**Figure A4:**
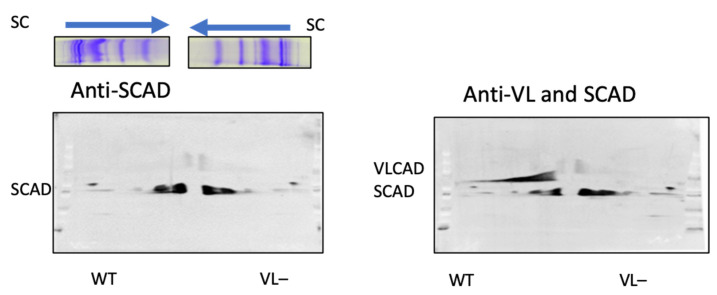
Additional uncut gel photos used for Figure 2 as marked.

**Figure A5:**
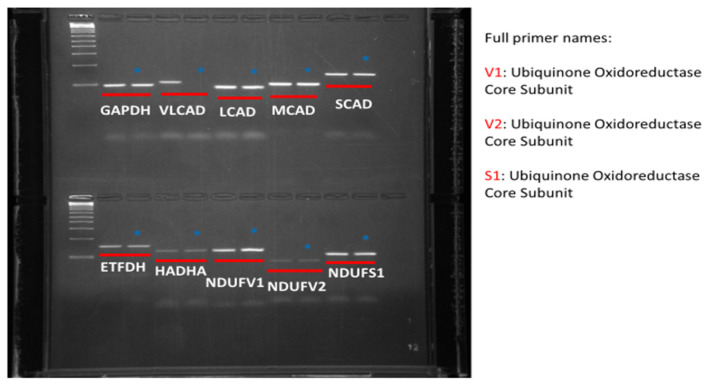
Uncut gel photos used for Figure 4 as marked.

**Figure A6:**
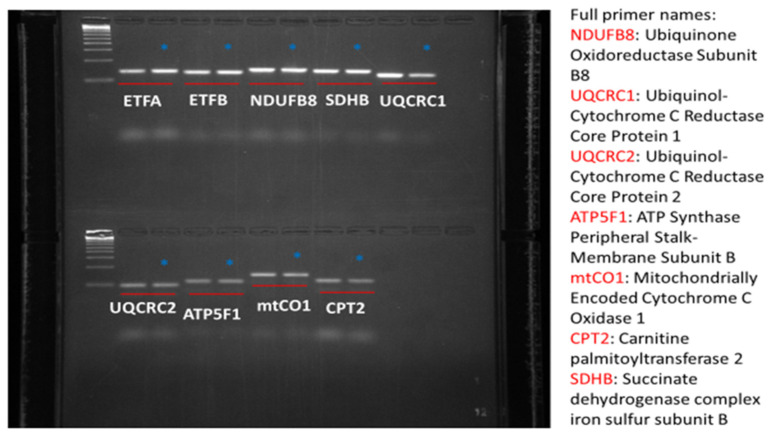
Additional uncut gel photos used for Figure 4 as marked.

## Data Availability

Primary data from this study are available from the corresponding author.

## Abbreviations

The following abbreviations are used in this manuscript:

FAO	Fatty acid β-oxidation
ETC	Electron transport chain
TCA	Tricarboxylic acid
SC	Supercomplex
ETC-SC	Electron transport chain supercomplex
MEC	Macromolecular energy complex
Com	Complex
ROS	Reactive oxygen species
CD	Circular dichroism spectroscopy
BNGE	Blue native polyacrylamide gel electrophoresis
VLCAD	Very-long-chain acyl-CoA dehydrogenase
TFP	Trifunctional protein
ETFDH	Electron flavoprotein dehydrogenase
SCAD	Short-chain acyl-CoA dehydrogenase
LCAD	Long-chain acyl-CoA dehydrogenase
MCAD	Medium-chain acyl-CoA dehydrogenase
CPT II	Carnitine palmitoyltransferase II
QH_2_	Coenzyme Q

## Appendix A

### Original Gels and Blots Used for All Figures as Detailed in the Figure Legends

**Table A1:** Reverse-transcription PCR primer list.

Primer Design of Reverse-Transcription PCR (RT-PCR) Analysis mRNA Content of FAO Genes in WT and VLCAD KO Mouse Heart Mitochondria		Primer Design of Reverse-Transcription PCR (RT-PCR) Analysis mRNA Content of ETC Genes in WT and VLCAD KO Mice Heart Mitochondria	
Gene (FAO)	Primer	Gene (ETC)	Primer
Mm_GAPDH_F	ACAGTCAAGGCCGAGAATGG	Mm_NDUFV1_F	CAAAGGAAGGAGGTCCCTG
Mm_GAPDH_R	GGCCTCACCCCATTTGATGT	Mm_NDUFV1_R	CTGTCACCTTCACAACTCCGA
Mm_VLCAD_F	TCTATGGCACAAAGGCCCAG	Mm_NDUFV2_F	TGAGGCATGGTTTGGCCATC
Mm_VLCAD_R	GGCTACATCGGATCCACTCG	Mm_NDUFV2_R	CAGCCTACCAACTCCACCAG
Mm_HADHA_F	CCCTTCAGTAAGGAAGCCTG	Mm_NDUFS1_F	CCGGAGGAAAGCCATCATGT
Mm_HADHA_R	GCAGTCAAGCATAAGGCATGG	Mm_NDUFS1_R	TACTTGCTGCTGTGCCAGTTG
Mm_ETFDH_F	TGTGACCCGGAAACCTTACTG	Mm_NDUFB8_F	GGAATCGTGTGGACACGTCA
Mm_ETFDH_R	CCATCACCTGCCAAGAAGCC	Mm_NDUFB8_R	GTACTGCTTCGGACCCACAG
Mm_LCAD_F	CTTGCTTGGCATCAACATCGC	Mm_SDHB_F	AGCTACTGGTGGAACGGAGA
Mm_LCAD_R	GAGTACGCTTGCTCTTCCCA	Mm_SDHB_R	GCAGCGGTAGACAGAGAAGG
Mm_MCAD_F	GAACTAAACATGGGCCAGCGA	Mm_UQCRC1_R	CGGATCCGGTTGTAGTCTGG
Mm_MCAD_R	GAAACCTGCTCCTTCACCGA	Mm_UQCRC2_F	GTCAGAGGGCTTCCTGAGTG
Mm_SCAD_F	TTGCCGAGAAGGAGTTGGTC	Mm_UQCRC2_R	AGCCACGGAGTCAATCTGTTG
Mm_SCAD_R	AGGTAATCCAAGCCTGCACC	Mm_ATP5F1_F	TCCAGGGGTATTACAGGCAAC
Mm_ETFA_F	GCTGGCTTTGTTCCCAATGAC	Mm_ATP5F1_R	TCAGGAATCAGCCCAAGACG
Mm_ETFA_R	TAGCCACGATTGTCTTGCTGT	Mm_mtCO1_F	TCGGAGCCCCAGATATAGCA
Mm_ETFB_F	AAAGCCGGACAAGTCTGGAG	Mm_mtCO1_R	TTTCCGGCTAGAGGTGGGTA
Mm_CPT2_F	GATGCTCCGAGGCATTTGTC		
Mm_CPT2_R	CCCATCGCTGCTTCTTTGGT		
